# The Clinical Details of *MYH9*-Related Disease and DFNA17 in a Large Japanese Hearing Loss Cohort

**DOI:** 10.3390/genes17020154

**Published:** 2026-01-29

**Authors:** Shinichi Goto, Akira Sasaki, Shin-ya Nishio, Chikako Shinkawa, Kiyoshi Oda, Tetsuro Wada, Kotaro Ishikawa, Tetsuo Ikezono, Shin-ichiro Oka, Nobuhiro Nishiyama, Taku Ito, Marina Kobayshi, Kozo Kumakawa, Naoko Sakuma, Hiroshi Nakanishi, Chihiro Morimoto, Natsumi Uehara, Testuya Okazaki, Kazuma Sugahara, Takeshi Nakamura, Shin-ichi Usami

**Affiliations:** 1 Department of Otorhinolaryngology Head and Neck surgery, Hirosaki University Graduate School of Medicine, Hirosaki 036-8560, Japan; goto-s@hirosaki-u.ac.jp (S.G.); akiras@hirosaki-u.ac.jp (A.S.); 2 Department of Hearing Implant Sciences, Shinshu University School of Medicine, Matsumoto 390-8621, Japan; nishio@shinshu-u.ac.jp; 3 Department of Otolaryngology, Head and Neck Surgery, Yamagata University Faculty of Medicine, Yamagata 990-9585, Japan; chikakoshinkawa@gmail.com; 4 Department of Otorhinolaryngology, Touhoku Rousai Hospital, Sendai 981-0911, Japan; oda.kiy@gmail.com; 5 Department of Otolaryngology-Head & Neck Surgery, Institute of Medicine, University of Tsukuba, Tsukuba 300-8575, Japan; twada@md.tsukuba.ac.jp; 6 Department of Otolaryngology, National Rehabilitation Center for Persons with Disabilities, Tokorozawa 359-8555, Japan; ishikawa-kotaro@rehab.go.jp; 7 Department of Otorhinolaryngology, Saitama Medical University, Moroyama 350-0495, Japan; ikezono.tetsuo@1972.saitama-med.ac.jp; 8 Department of Otorhinolaryngology, International University of Health and Welfare, Mita Hospital, Tokyo 108-8329, Japan; okashin@shinshu-u.ac.jp; 9 Department of Otorhinolaryngology-Head and Neck Surgery, Tokyo Medical University, Tokyo 160-0023, Japan; nobuoto@tokyo-med.ac.jp; 10 Department of Otorhinolaryngology, Institute of Science Tokyo, Tokyo 113-8510, Japan; taku.oto@tmd.ac.jp; 11 Department of Otorhinolaryngology, Toranomon Hospital, Tokyo 105-8470, Japan; marinakobayashi@nms.ac.jp; 12 Department of Otorhinolaryngology, Akasaka Toranomon Clinic, Tokyo 107-0052, Japan; kozo3000@gmail.com; 13 Department of Otorhinolaryngology, Head and Neck Surgery, Nippon Medical School, Tokyo 113-8603, Japan; naoko-sakuma@nms.ac.jp; 14 Department of Otorhinolaryngology/Head and Neck Surgery, Hamamatsu University School of Medicine, Hamamatsu 431-3192, Japan; hiro-na@hama-med.ac.jp; 15 Department of Otolaryngology-Head and Neck Surgery, Nara Medical University, Kashihara 634-8522, Japan; mori-chi@naramed-u.ac.jp; 16 Department of Otolaryngology-Head and Neck Surgery, Kobe University Graduate School of Medicine, Kobe 650-0017, Japan; natsuko@med.kobe-u.ac.jp; 17 Department of Medical Genetics, Tottori University School of Medicine, Yonago 683-8504, Japan; t-okazaki@tottori-u.ac.jp; 18 Department of Otolaryngology, Yamaguchi University Graduate School of Medicine, Ube 755-8505, Japan; kazuma@yamaguchi-u.ac.jp; 19 Department of Otolaryngology, Faculty of Medicine, University of Miyazaki, Miyazaki 889-1692, Japan; takeshi_nakamura@med.miyazaki-u.ac.jp

**Keywords:** *MYH9*, progressive hearing loss, DFNA17, *MYH9*-related disease, cochlear implantation

## Abstract

**Background/Objectives**: *MYH9* gene variants cause *MYH9*-related disease (*MYH9*-RD), which is also known as Epstein syndrome, Fechtner syndrome, May–Hegglin anomaly, and Sebastian syndrome. *MYH9*-RD is characterized by sensorineural hearing loss, macrothrombocytopenia, thrombocytopenia, hematuria/proteinuria, glomerulonephritis, cataracts purpura, and mucosal bleeding. In addition, the *MYH9* gene is also known to be causative of autosomal dominant non-syndromic hearing loss (DFNA17). *MYH9*-RD is a relatively rare disorder, and the detailed clinical features and mutational spectra remain unclear. **Methods**: In this study, we performed next-generation sequencing analysis for 15,684 hearing loss patients and identified *MYH9*-associated hearing loss patients. Detailed clinical information was collected for these patients and summarized. **Results**: In this study, we identified 24 patients from 18 families with *MYH9*-associated hearing loss. We clarified the details of hearing deterioration observed in patients based on collected serial audiogram data. Some cases showed rapid hearing deterioration that worsened by about 50 dB within 5 years. Hearing loss is more likely to progress in patients with myosin head domain variants than in patients with myosin tail domain variants, but hearing loss in each set of patients finally deteriorates to bilateral profound hearing loss. **Conclusions**: In this study, we were able to clarify the detailed characteristics of *MYH9*-RD- and DFNA17-related hearing loss in a relatively large number of patients, particularly in some cases that showed rapid and asymmetrical hearing deterioration progressing to bilateral profound hearing loss. Our data will be useful for providing more appropriate treatment and follow-up for *MYH9*-associated hearing loss.

## 1. Introduction

Hearing loss (HL) is one of the most frequent sensory disorders, with more than 150 genes identified as causes of HL [1]. Identification of the causative gene of HL enables us to estimate the type of HL, the progressiveness of HL, and the associated symptoms. Based on the epidemiological analysis, 80% of cases of congenital HL are non-syndromic, whereas the remaining 20% are syndromic and characterized by HL with associated symptoms [2]. Most cases of syndromic HL can be diagnosed based on the combination of symptoms; however, it is sometimes difficult to diagnose patients based only on clinical phenotype, particularly in cases with late-onset symptoms or an incomplete combination of symptoms.

*MYH9* gene (MIM *160775) variants are known to cause either autosomal dominant syndromic HL known as *MYH9*-related disease (*MYH9*-RD, MIM #155100) [3] or autosomal dominant non-syndromic HL (DFNA17, MIM #603622) [4]. *MYH9*-RD was first reported as four different syndromes: Epstein syndrome, Fechtner syndrome, May–Hegglin anomaly, and Sebastian syndrome. However, after genetic analysis, all these syndromes were found to be caused by genetic variants in the *MYH9* gene and came to be regarded as a single spectrum disorder. The characteristic clinical feature of *MYH9*-RD is congenital macrothrombocytopenia. This macrothrombocytopenia is characterized by a triad of large platelets, thrombocytopenia, and distinctive Döhle body-like leukocyte inclusions [5]. Most cases develop late-onset sensorineural hearing loss (SNHL), protein-uric nephropathy, presenile cataract, and/or alteration of liver enzymes [3,6]. Sensorineural hearing loss (SNHL) is observed in about half of the patients, and while some patients show mild-to-moderate SNHL, others show progressive HL that finally deteriorates to severe-to-profound HL [7,8]. The patients with severe-to-profound SNHL tend to have early-onset HL [9].

The *MYH9* gene is located on 22q12.3 and encodes the non-muscle myosin heavy chain IIA (NMMHC-IIA) [10,11]. The *MYH9* gene contains 41 exons and consists of an N-terminal head domain and a C-terminal tail domain. NMMHC-IIA expresses most cell types and tissues, including granulocytes, megakaryocytes, and glomerular epithelial cells [12]. According to studies using rodent inner ears, NMMHC-IIA is also expressed predominantly in the hair cells of the stereocilia, spiral ligament, and spiral limbus [13].

To date, 240 *MYH9* gene variants have been reported as pathogenic variants [14]. These previous studies have described mainly the clinical phenotypes of macrothrombocytopenia, with few reports focusing on SNHL. Therefore, the detailed characteristics of SNHL, such as its progressiveness or severity, remain unclear. In addition, the genotype–phenotype correlations associated with SNHL remain to be clarified. In this study, we aimed to elucidate the variant spectrum of the *MYH9* gene and obtain a more precise description of the features associated with *MYH9*-associated HL.

## 2. Materials and Methods

### 2.1. Subjects

For this study, a total of 15,684 Japanese HL patients were enrolled from 102 otolaryngology departments nationwide, as described in our previous reports [15]. Written informed consent was obtained from all patients (or from their next of kin, caretaker, or legal guardian in case of minors or children). The Shinshu University Ethical Committee as well as the respective Ethical Committees of the other participating institutions approved this study. This study was conducted in accordance with the Declaration of Helsinki and the protocol was approved by the Ethics Committee of Shinshu University School of Medicine (No.387—4 September 2012, No.576—2 May 2017 and No.718—7 March 2022).

### 2.2. Clinical Evaluations

The onset age of HL, family history, the degree of progressiveness, and associated symptoms (including macrothrombocytopenia, leukocyte inclusion, hematuria, proteinuria, glomerulonephritis, cataracts, purpura, and mucosal bleeding) were analyzed based on the medical charts of the probands and their family members harboring the same *MYH9* variants. Pure-tone average (PTA) was calculated from the audiometric thresholds at four frequencies (0.5, 1, 2, and 4 kHz). The severity of HL was classified into five categories: mild HL (>25 dB and ≤40 dB HL), moderate HL (>40 dB and ≤70 dB HL), severe (>70 dB and ≤90 dB HL), and profound (>90 dB HL). Asymmetric HL was defined as a difference in PTA of over 20 dB between the right and left ears. The audiometric configurations were categorized into low-frequency, mid-frequency (U-shaped), high-frequency, flat type, and deaf, as reported previously [16]. Statistical analysis was performed using Welch’s *t*-test. Missing data were removed from the analysis.

### 2.3. Next-Generation Sequencing and Bioinformatic Analysis

An amplicon library was prepared with an Ion AmpliSeq Library Kit 2.0 (ThermoFisher Scientific, Waltham, MA, USA) and Ion AmpliSeq^TM^ Custom Panel (ThermoFisher Scientific) for 158 target genes reported to cause non-syndromic HL and syndromic HL [17]. After the amplicon libraries were prepared, next-generation sequencing analysis was performed using an Ion S5 plus instrument with an Ion 540 Kit-Chef and Ion 540 Chip Kit (ThermoFisher Scientific) according to the manufacturer’s instructions. The sequence data mapping was performed against the human genome sequence (build GRCh37/hg19) using the Torrent Mapping Alignment Program. After sequencing mapping, the DNA variant regions were piled up with Torrent Variant Caller plug-in software ver. 5.16. The impacts of the variants were investigated using ANNOVAR software ver. 2020-06-08 [18]. Variants were further selected as <1% of minor allele frequencies (MAFs) in several control databases, including the 1000 genome database (http://www.1000genomes.org/), The Genome Aggregation Database (https://gnomad.broadinstitute.org), the 60,000 Japanese genome variation database ToMMo 60KJPN (https://jmorp.megabank.tohoku.ac.jp/), and the 333 in-house Japanese normal hearing controls. All filtering was carried out using our original database software, as previously reported [19]. Direct Sanger sequencing was performed to confirm the identified variants and family segregation analysis. The pathogenicity of the identified variants was evaluated in accordance with the American College of Medical Genetics (ACMG) standards and guidelines [20] and the ClinGen Hearing Loss Clinical Domain Working Group expert specification [21]. The variants classified as “Likely Pathogenic” or “Pathogenic” in the ACMG guidelines were considered to be causative variants. Furthermore, variants identified as being of “Uncertain Significance” were also considered to be causal if all of the following requirements were satisfied: (1) no other candidate variants were identified in the other 157 genes; (2) the MAF was extremely low in the control populations in gnomAD, ToMMo 60KJPN, and in-house controls; (3) the pathogenic impact was supported by the majority of the in silico prediction scores; (4) no contradictory evidence exists regarding the pathogenicity of the detected candidate variant.

## 3. Results

### 3.1. Identified Variants

As a result of the next-generation sequencing analysis for the large HL Japanese cohort, we identified 13 possibly disease-causing *MYH9* variants in 18 independent families with HL. The variants identified in this study are summarized in Table 1. Among the thirteen variants, four variants were novel and nine variants were previously reported as causative for *MYH9*-associated HL. Six variants were located in the myosin head domain (HD) and four were located in the myosin tail domain (TD). All novel variants identified in this study were missense or non-frameshift insertion variants, and these variants were classified as variants of “Uncertain Significance” based on the ACMG guidelines. However, we regarded these variants as candidate causative variants for *MYH9*-associated HL as they fulfilled the criteria described in the Methods Section. Most of the in silico pathogenicity prediction scores supported the pathogenicity of the identified variants. In addition, MAFs in the control populations fulfilled the criteria supporting their pathogenicity defined by the ClinGen HL expert panel (MAF ≤ 0.00002). However, all novel variants were of “Uncertain Significance,” and further studies are needed to confirm the pathogenicity of those variants.

### 3.2. Characteristics of MYH9-Associated Hearing Loss and Outcomes of Cochlear Implantation

**Figure 1:**
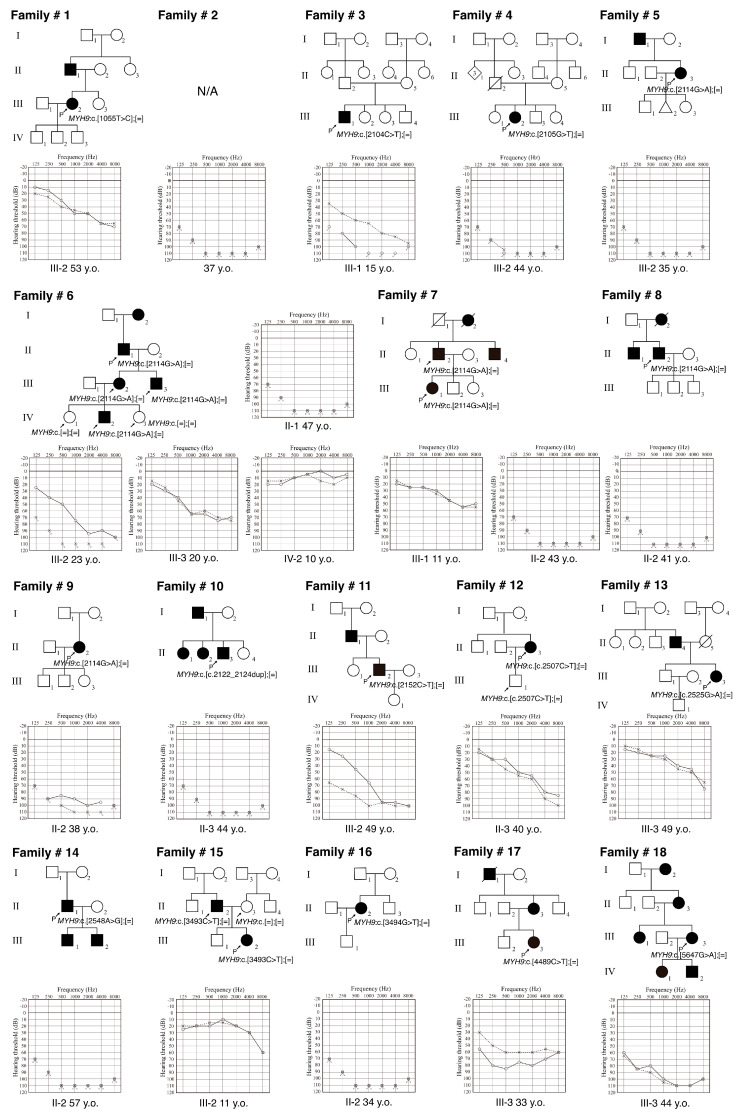
Pedigrees and audiograms of the 20 families who carried *MYH9* variants identified in this study. Filled symbols indicate affected individuals. Arrows indicate the individuals who underwent genetic testing. Arrows with P symbols indicate the probands in each family. Circles indicate hearing thresholds for the right ear and crosses show hearing thresholds for the left ear.

The pedigrees and audiograms of the *MYH9*-associated HL patients identified in this study are shown in Figure 1, and clinical characteristics are summarized in Table 2. In this study, we identified 24 patients with *MYH9*-associated HL from 18 independent families. A total of ten patients were male, and fourteen patients were female. Among the 18 families, 13 had an autosomal dominant (AD) family history, while 4 were sporadic cases without any affected family member. Regarding the onset age of the HL, 4 patients had prelingual onset HL (below the age of 6) and 20 patients had post-lingual onset HL. The post-lingual onset age ranged widely from 6 to 45 years. The hearing levels at the examination varied from mild to profound. Ten patients had profound HL, three patients had severe HL, and nine patients had mild-to-moderate HL. Only two patients under 10 years of age (son of JHLB-9870 and grandson of JHLB-3212) have normal hearing while harboring the *MYH9* variant, although other family members with the same variant showed moderate-to-profound HL. Most of the patients with mild-to-moderate HL showed high-frequency sloping-type HL. We found that 91% (20/22) of patients were aware of their hearing loss progression and almost half of the patients had tinnitus (56.5%, 13/23). On the other hand, only 13% (3/23) of patients had a history of vertigo.

**Figure 2:**
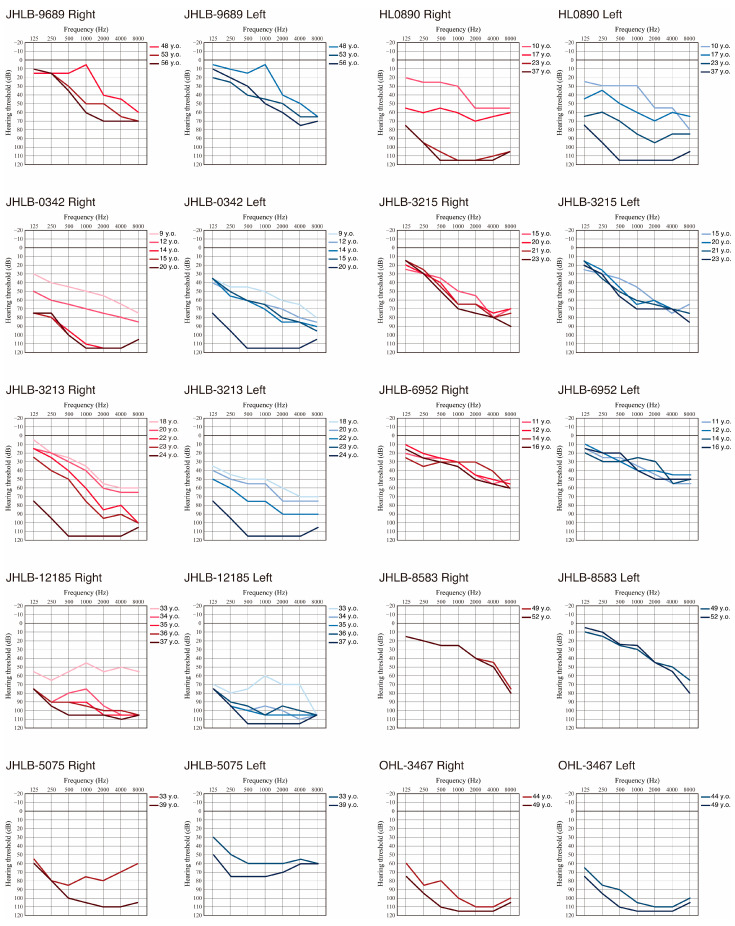
Overlapping audiograms of each *MYH9*-associated HL patient identified in this study. Red indicates hearing thresholds for the right ear and blue indicates hearing thresholds for the left ear. Darker coloring indicates hearing thresholds measured at older ages.

To clarify the hearing deterioration of *MYH9*-associated HL, we collected serial audiograms for 10 patients. As shown in Figure 2, eight cases, excluding JHLB-6952 and JHLB8583, showed hearing deterioration within the observation period. It is noteworthy that several cases showed asymmetrical hearing deterioration (see the 17 and 23 y.o. hearing thresholds for JHLB-0890, the 14 and 15 y.o. hearing thresholds for JHLB-0342, the 18 -23 y.o. hearing thresholds for JHLB-3213, the 33 and 34 y.o. hearing thresholds for JHLB-12185, and the 33 and 39 y.o. hearing thresholds for JHLB-5075 in Figure 2), finally deteriorating to bilateral profound HL. In addition, HL0890, JHLB-0342, JHLB-3213, and JHLB12185 showed rapid hearing deterioration, which worsened by about 50 dB (ranging from 48 dB to 67 dB) within five years. The detailed mechanism underlying this rapid hearing deterioration remains unclear, but careful follow-up is necessary for *MYH9*-associated HL patients due to the potential for rapid hearing deterioration.

**Figure 3:**
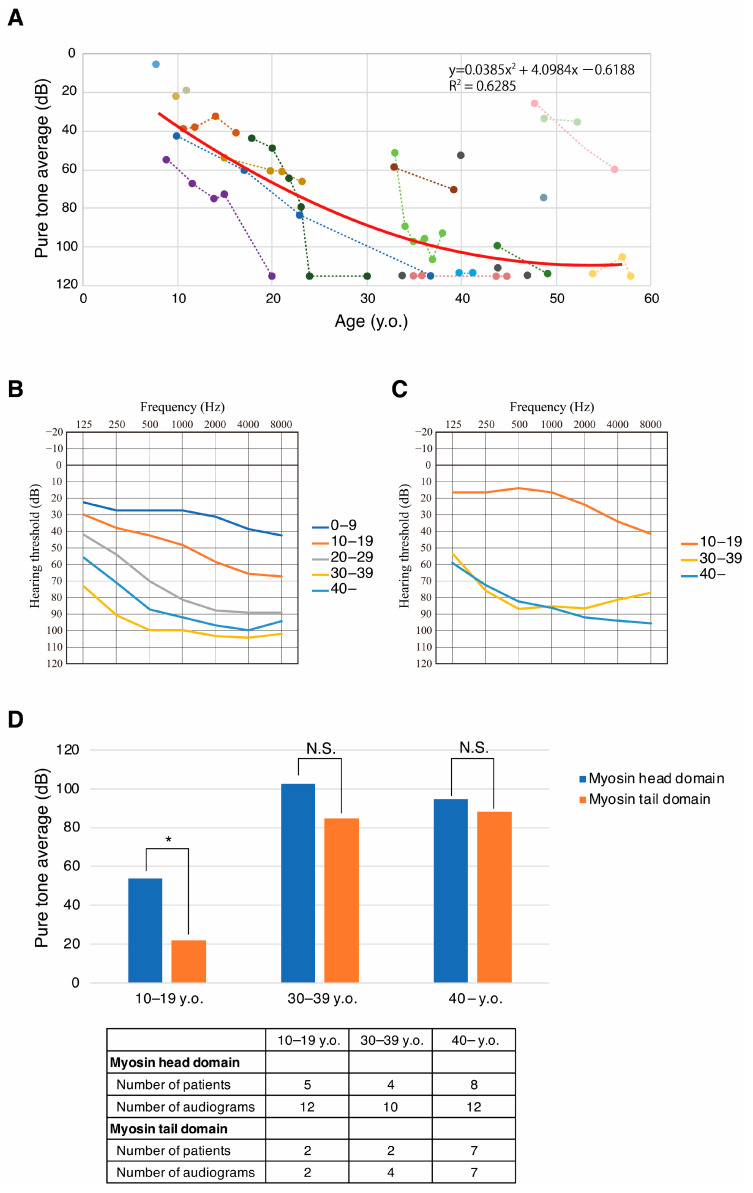
Scatter plotting of pure-tone average vs. age, and averaged audiogram for myosin head and tail domain variants. Several patients showed asymmetrical hearing deterioration; thus, this figure used the average of the left and right ear hearing thresholds. (**A**) Scatter plotting of pure-tone average (500 Hz, 1000 Hz, 2000 Hz, and 4000 Hz) and age of *MYH9*-associated HL patients identified in this study. Each different color indicates the hearing threshold for a different patient. Dashed lines indicate the hearing threshold change for the same patient. The red line is the regression line drawn without 2 cases who presented with mild HL even after age 45 y.o. (**B**) Mean hearing thresholds for each 10 years of age for the patients with myosin head domain variants. (**C**) Mean hearing thresholds for each 10 years of age for the patients with myosin tail domain variants. (**D**) Average PTA for the patients with myosin head domain variants and myosin tail domain variants by age group: 10–19 y.o., 30–39 y.o., and ≥40 y.o. We used all available audiometric data in this analysis and show the number of patients and number of audiograms used in this analysis at the bottom of the figure. Statistical analysis was performed by Welch’s *t*-test. *: statistically significant different. N.S.: not significant.

Scatter plotting of hearing thresholds and audiometric testing age is shown in Figure 3A. As shown in Figure 3A, the hearing threshold for most patients showed hearing deterioration with age, with most cases showing profound HL after 40 years of age. In addition, we compared mean hearing thresholds every 10 years for the patients carrying HD variants and those carrying TD variants (Figure 3B,C). As shown in Figure 3B,C, *MYH9*-associated HL patients carrying HD variants showed a stronger tendency for rapid hearing deterioration compared to those carrying TD variants. We also compared the PTA for the patients carrying HD variants and those carrying TD variants (Figure 3D), and found that patients with HD variants had more statistically severe HL than patients with TD variants in the 10–19 y.o. age group (*p* = 0.00027, Welch’s *t*-test), but not in the 30–39 y.o. or ≥40 y.o. age groups. However, the number of patients in the 10–19 y.o. age group was small and further investigation is still needed.

**Figure 4:**
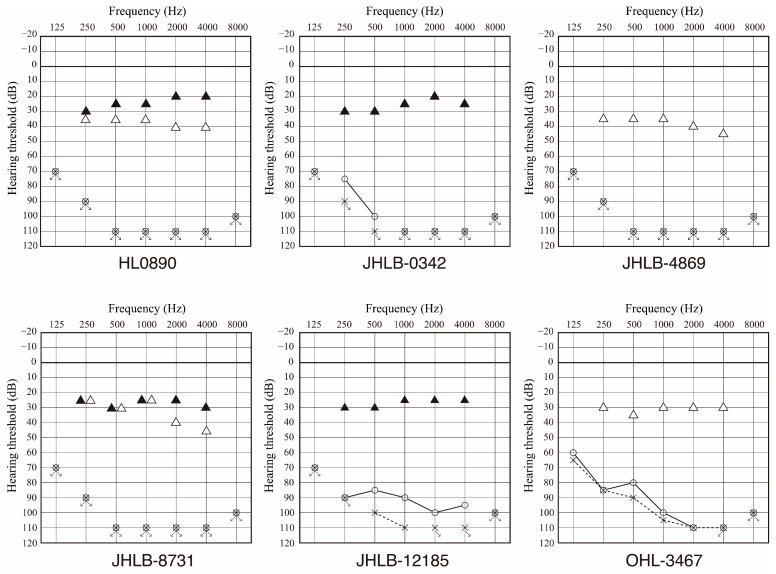
Hearing thresholds pre-CI surgery and hearing thresholds after cochlear implantation for the patients with *MYH9*-associated HL identified in this study. All six cases showed profound HL pre-CI and significant improvements were observed (about 30 dB in hearing level) with CI use. Filled triangles show hearing thresholds with CI for the right ear and open triangles show hearing thresholds with CI for the left ear. Circles indicate pre-CI hearing thresholds for the right ear and crosses show pre-CI hearing thresholds for the left ear.

Nine patients received cochlear implantation (CI). We obtained hearing thresholds after CI for six patients and found that the averaged hearing threshold was 30.5 dB (range 22.5 to 38.8 dB) (Figure 4). Average pre-CI hearing level in PTA for the same six patients was 108.1 dB (range 95 to 110 dB), and CI improved hearing level significantly (*p* = 2.3 × 10^−13^, Welch’s *t*-test). In addition, monosyllable perception score in a quiet setting was 75% (range 60 to 100%) on average. Thus, CI is a good treatment option for the patients with *MYH9*-associated HL.

### 3.3. Associated Symptoms of MYH9-Associated HL Patients Identified in This Study

The clinical features are shown in Table 2. We obtained clinical histories for thrombocytopenia from 18 patients, and found that 7 patients had thrombocytopenia (7/18; 38.9%) and 8 patients had macrothrombocytopenia (8/13; 61.5%). We obtained data regarding leukocyte inclusion from only four patients, and found that only one patient had leukocyte inclusion (1/4; 25%). Four patients had purpura (4/15; 26.7%) and four patients had mucosal bleeding (4/14; 28.6%).

We obtained clinical histories for hematuria or proteinuria from 13 patients, and found that 4 patients had hematuria/proteinuria (4/13; 30.8%). Furthermore, three patients had glomerulonephritis (3/12; 25.0%) and two of the three patients had hematuria/proteinuria. We also obtained clinical histories for cataracts from 14 patients, and found that only 1 patient had cataracts (1/14; 7.1%).

## 4. Discussion

The *MYH9* gene encodes myosin heavy chain 9 non-muscle, which is known as non-muscle myosin-IIA [10,11]. The pathogenic variants in the *MYH9* gene cause *MYH9*-RD or DFNA17 [3,4]. In this study, we identified 13 variants in 24 HL patients from 18 different families. Thus, the prevalence of *MYH9*-associated HL in this study was 0.11% (18/15,684) in Japanese HL patients.

**Figure 5:**
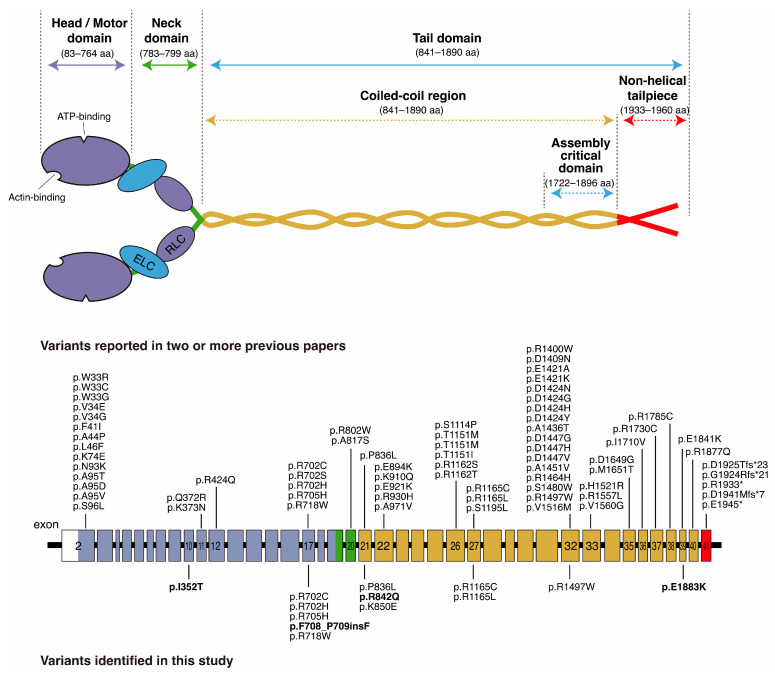
Domain structure illustration for NMIIA protein, exon structure, and variants identified in this study and previous papers. Purple indicates the myosin head domain, green indicates the neck domain, yellow indicates the coiled-coil domain, and red indicates the non-helical tailpiece domain. Previously reported variants were restricted to the variants which were reported in two or more previous papers. Bold indicates the novel variants identified in this study. This figure was prepared with reference to the previous reports [30,31].

Among the 13 variants, 6 were HD and 6 were TD variants (Figure 5). It has been reported that patients with pathogenic variants in the HD have a higher risk of early-onset and severe deafness than patients with variants in the TD [7,28,29]. Pecci et al. reported that amino acid substitutions of arginine 702 of the HD are related to the most severe phenotype: all individuals with mutations involving this residue experience end-stage renal disease and profound HL before the fourth decade [28]. On the other hand, p.Asp1424His variants in the coiled-coil region are associated with late-onset symptoms. This variant disrupts the interactions between the SH3 motif and the 50 kDa sub-domain of the HD and increases the risk of deafness while decreasing the risk of kidney disorders or cataracts. Variants involving the arginine 1165 residue in the coiled-coil region also showed a similar phenotype [30]. In this study, patients with variants in either the HD or the TD demonstrated progressive HL (Figure 3B,C). Similarly to previous reports, our patients with variants in the HD showed a tendency for more severe HL compared with the patients with TD variants (Figure 3B–D). However, the number of patients in each age group used in this study was relatively small and further investigation is still needed.

Pecci et al. reported that the HL types in *MYH9*-associated HL patients were mostly sloping-type [8]. In the cases with DFNA17, a mild-to-moderate sloping-type HL is first observed, followed by deterioration of the low-frequency hearing level and eventual progression to severe, flat-type HL [32]. We observed a similar progression in hearing loss to that reported in previous studies (Figure 2).

Nine patients received CI with favorable outcomes. In previous studies, several patients with *MYH9*-RD underwent CI and almost all of the patients had effective outcomes with better postoperative speech perception scores after CI [8,28,33,34,35]. According to studies on the inner ear of rodents, NMMHC-IIA is expressed in the hair cells of the stereocilia, spiral ligament, and spiral limbus [13]. Good CI performance can be expected because *MYH9* expression is limited to the hair cells of the stereocilia, spiral ligament, and spiral limbus, and the spiral ganglions appear to remain intact. In addition, it is important to manage bleeding during CI surgery for patients with thrombocytopenia. In our study, we obtained information regarding CI surgery in one patient, and found that the surgery was performed without major bleeding complications. On the other hand, there have been reports of cases in which platelet transfusion was performed preoperatively to prevent perioperative bleeding [34,35]. Mori et al. reported a case where eltrombopag, a nonpeptide agonist of the thrombopoietin receptor, was used, meaning that they could avoid using platelet transfusion [33]. Therefore, CI is a safe procedure in *MYH9*-RD patients whenever adequate preventative measures are taken.

In our study, the prevalence of thrombocytopenia was low (38.9%: 7/18) compared to that in previous studies [8,28,36,37]. Similarly, the prevalence of mucosal bleeding was also low (28.6%: 4/14). Eight patients were observed to have macrothrombocytopenia (61.5%: 8/13). In the case of macrothrombocytopenia, automated cell counters usually underestimate platelet counts because platelets are mainly identified based on size [38]. It is important to avoid unnecessary blood transfusions when performing CI surgery in such cases. In previous studies, all patients with *MYH9* variants had macrothrombocytopenia, which differs from the findings in this study [8,28]. Leukocyte inclusions involving Döhle-like bodies were present in 42–84% of *MYH9*-RD patients [3,39,40]. However, there was only one patient with leukocyte inclusion in our study. It is noteworthy that there were a variety of clinical phenotypes associated with thrombocytopenia or macrothrombocytopenia, even in the patients carrying the same variant (e.g., patients carrying the c.2114G > A variant). In a previous study, the c.2114G > A variant was also identified from non-syndromic hearing loss (DFNA17) patients [31]. On the other hand, the prevalences of hematuria/proteinuria (30.8%: 4/13), glomerulonephritis (25.0%: 3/12), and cataracts (7.5%: 1/14) were comparable to those in previous reports [8,28].

In this study, we mainly analyzed non-syndromic HL patients from collaborating otolaryngology departments nationwide, so we could access not only *MYH9*-RD but also DFNA11 patients who did not show any associated symptoms. However, to avoid the possibility of overlooking associated symptoms, we need to collaborate with other departments, such as hematology and nephrology departments, in future studies.

## 5. Conclusions

In this study, we were able to clarify the detailed characteristics of HL associated with *MYH9*-RD and DFNA17 in a relatively large number of patients. One of the remarkable results of this study was our ability to clarify the details of hearing deterioration using serial audiogram data collected from the same patients. Similarly to previous reports, patients with HD variants showed a tendency for relatively severe HL in comparison to those with TD variants, but both cases deteriorated to profound HL. In addition, we showed that CI was an effective treatment option for patients with severe-to-profound HL. The results identified in this study will be beneficial in enabling more appropriate treatment for patients with *MYH9*-associated HL based on the expectation of future hearing deterioration and favorable CI outcomes.

## Figures and Tables

**Table 1:** *MYH9* variants identified in this study.

Nucleotide Change	AA Change	Domain	SIFT	PP2	MutTaster	REVEL	CADD	ToMMo 60KJPN	gnomAD All	Pathogenicity	Reference
c.1055T > C	p.Ile352Thr	Myosin head	D	B	D	0.878	29.3	0	0.0000007	Uncertain Significance	This study
c.2104C > T	p.Arg702Cys	Myosin head	D	D	A	0.884	35	0	0	Pathogenic	Seri M et al., 2000 [11]
c.2105G > A	p.Arg702His	Myosin head	D	D	A	0.884	35	0	0.0000066	Pathogenic	Heath KE et al., 2001 [22]
c.2114G > A	p.Arg705His	Myosin head	D	D	A	0.829	35	0	0	Pathogenic	Lalwani AK et al., 2000 [4]
c.2122_2124dup	p.Phe708_Pro709insPhe	Myosin head	.	.	.	.	.	0	0	Uncertain Significance	This study
c.2152C > T	p.Arg718Trp	Myosin head	D	D	D	0.776	34	0	0.0000007	Pathogenic	Pecci A et al., 2008 [23]
c.2507C > T	p.Pro836Leu	.	D	D	D	0.963	34	0.0000080	0.0000014	Uncertain Significance	Neveling K et al., 2013 [24]
c.2525G > A	p.Arg842Gln	Myosin tail	D	B	D	0.441	31	0	0	Uncertain Significance	This study
c.2548A > G	p.Lys850Glu	Myosin tail	D	P	D	0.870	32	0	0	Pathogenic	Saposnik B et al., 2014 [25]
c.3493C > T	p.Arg1165Cys	Myosin tail	D	D	A	0.921	34	0	0	Pathogenic	Seri M et al., 2000 [11]
c.3494G > T	p.Arg1165Leu	Myosin tail	D	P	A	0.924	34	0	0	Pathogenic	Kunishima S et al., 2001 [26]
c.4489C > T	p.Arg1497Trp	Myosin tail	D	D	D	0.796	35	0	0.0000041	Uncertain Significance	Sloan-Heggen CM et al., 2016 [27]
c.5647G > A	p.Glu1883Lys	Myosin tail	D	D	D	0.847	35	0	0.0000021	Uncertain Significance	This study

All variants are indicated on NM_002473. AA: amino acid; PP2: PolyPhen2; MutTaster: Mutation Taster; D (in SIFT): deleterious; D (in PP2): probably damaging; P: possibly damaging; B: benign; D (in MutTaster): disease causing; A: disease-causing automatic.

**Table 2:** Clinical characteristics of *MYH9*-associated hearing loss patients identified in this study.

Family Number	ID	AA Change	Hereditary	Onset	Age	Gender	Severity of HL	Type of HL	Fluctuation of HL	Progression of HL	Thrombocytopenia	Macrothrombocytopenia	Hematuria/Proteinuria	Glomerulonephritis	Cataracts	Purpura	Mucosal Bleeding
1	JHLB-9689	p.Ile352Thr	AD	45	53	F	Moderate	Sloping	+	+	-	NA	NA	+	-	NA	NA
2	HL0890	p.Arg702Cys	NA	2	37	F	Profound	Flat	NA	+	+	+	+	+	+	+	+
3	JHLB-0342	p.Arg702Cys	Sporadic	3	15	M	Severe	Sloping	+	+	+	+	+	-	-	+	+
4	JHLB-4869	p.Arg702His	Sporadic	17	44	F	Profound	Flat	-	+	+	+	+	+	-	-	-
5	JHLB-1380	p.Arg705His	AD	10	35	F	Profound	Flat	-	+	NA	NA	NA	NA	NA	NA	NA
6	JHLB-3212	p.Arg705His	AD	26	47	M	Profound	Flat	+	+	-	NA	-	-	-	-	-
	JHLB-3215 (Son)	p.Arg705His		6	20	M	Moderate	Sloping	+	+	-	NA	-	-	-	-	-
	JHLB-3213 (Daughter)	p.Arg705His		5	23	F	Moderate	Sloping	+	+	+	+	-	-	-	-	+
	JHLB-14696 (Grandson)	p.Arg705His		10	10	M	Normal	Flat	+	+	NA	NA	NA	NA	NA	NA	NA
7	JHLB-6952	p.Arg705His	AD	8	11	F	Mild	Sloping	-	+	-	-	NA	NA	-	+	+
	JHLB-7171 (Father)	p.Arg705His		17	43	M	Profound	Flat	+	+	-	-	NA	NA	-	-	-
8	JHLB-8731	p.Arg705His	AD	15	41	M	Profound	Flat	+	+	-	-	-	NA	-	-	-
9	JHLB-12185	p.Arg705His	AD	26	38	F	Severe	Flat	-	+	-	+	-	NA	NA	-	-
10	JHLB-11594	p.Phe708_Pro709insPhe	AD	30	44	M	Profound	Flat	-	+	NA	NA	NA	NA	NA	NA	NA
11	JHLB-15848	p.Arg718Trp	AD	20	49	M	Severe	Sloping	-	+	NA	NA	NA	NA	NA	NA	NA
12	JHLB-9870	p.Pro836Leu	Sporadic	22	40	F	Moderate	Sloping	-	+	-	+	NA	-	NA	-	-
	JHLB-15035 (Son)	p.Pro836Leu		2	2	M	Normal	Flat	-	-	-	+	-	-	-	-	-
13	JHLB-8583	p.Arg842Gln	AD	18	49	F	Mild	Sloping	-	+	NA	NA	NA	NA	NA	NA	NA
14	JHLB-13047	p.Lys850Glu	AD	5	57	M	Profound	Flat	-	+	+	NA	+	NA	NA	+	NA
15	JHLB-14621	p.Arg1165Cys	AD	10	11	F	Mild	Sloping	-	NA	NA	NA	NA	NA	NA	NA	NA
	JHLB-15737 (Father)	p.Arg1165Cys		40	45	M	Mild	Sloping	-	-	+	NA	NA	NA	-	NA	NA
16	JHLB-14856	p.Arg1165Leu	Sporadic	34	34	F	Profound	Flat	NA	NA	+	+	-	-	NA	NA	NA
17	JHLB-5075	p.Arg1497Trp	AD	9	33	F	Moderate	Flat	+	+	-	-	-	-	-	+	-
18	OHL-3467	p.Glu1883Lys	AD	36	44	F	Profound	Flat	-	+	-	-	-	-	-	-	-

HL: hearing loss; AD: autosomal dominant; M: male; F: female; NA: data not available.

## Data Availability

The datasets used during the current study are available from the corresponding author on reasonable request.
